# 

**Published:** 1899-07-22

**Authors:** 


					The HOSPITAL. " The standard of highest purity."?Lancet. July 22nd, 1899. ?
cadbury's rt H m CADo!cUo?YS
COGOA
ABSOLUTELY PURS.
NO DRUGS OR ALKALIES USED.
No. 66V Vol. XXVI. rssa Saturday,
July 22nd, 1899.
L see p. 274.
I rxwiuli x W UJT Xi JM vLi

				

